# Silent synapses in pain-related anterior cingulate cortex

**DOI:** 10.1177/17448069231179011

**Published:** 2023-05-25

**Authors:** Min Zhuo

**Affiliations:** 1 Department of Pharmacology, Qingdao University School of Pharmacy, Qingdao, China; 2 Department of Neurology, First Affiliated Hospital of Guangzhou Medical University, Guangzhou, China; 3 Department of Physiology, Faculty of Medicine, 7938 University of Toronto, Toronto, ON, Canada

**Keywords:** Chronic pain, adult anterior cingulate cortex, silent synapses, glutamate, AMPA receptors, NMDA receptors

## Abstract

Synaptic plasticity such as Long-term potentiation (LTP) is a key mechanism for learning in central synapses including the cortex. There are two least two major forms of LTPs: presynaptic LTP and postsynaptic LTP. For postsynaptic LTP, the potentiation of AMPA receptor-mediated responses through protein phosphorylation is thought to be a key mechanism. Silent synapses have been reported in the hippocampus, but it is thought to be mainly present in the cortex during early development, and may contribute to maturation of the cortical circuit. However, recent several lines of evidence demonstrate that silent synapses may exist in mature synapses of adult cortex, and they can be recruited by LTP-inducing protocols, as well as chemical-induced LTP. In pain-related cortical regions, silent synapses may not only contribute to cortical excitation after peripheral injury, but also the recruitment of new cortical circuits as well. Thus, it is proposed that silent synapses and modification of functional AMPA receptors and NMDA receptors may play important roles in chronic pain, including phantom pain.

## Introduction

Long-term potentiation (LTP) is a key cellular mechanism for our understanding of physiological and pathological mechanisms of learning and memory, chronic pain, emotional disorders, drug addiction and neuronal development.^1–7^ Synaptic learning not only contributes to behavioral learning and memory, but also plays important roles in long-term neuronal changes associated with many other functions such as chronic pain, fear and depression. The study of synaptic plasticity therefore is essential for our understanding of almost most of brain key functions.

The study of synaptic mechanism in the hippocampus, especially CA1 region, lead major progress of our understanding of synaptic transmission and plasticity.^8–10^ Among several possible mechanisms that contribute to the formation of LTP, silent receptors have caused much of attention. It serves as one of the most direct evidence for postsynaptic mechanism for the expression of LTP. Our understanding of basic mechanisms of memory has also greatly improved our understanding of synaptic mechanisms in many chronic diseases such as chronic pain.^1,3,4^ In this review, I will summarize recent work of silent synapses in pain-related cortex; and propose that it may exist as a key synaptic mechanism for cortical circuit LTP and spreading in chronic pain.

## Silent synapses and their contribution to LTP

Silent synapses were first reported in young hippocampal neurons in the CA1 region.^8,10^ Silent synapses are the synapses that lack functional receptors to detect the release of presynaptic transmitter such as glutamate. At silent glutamatergic synapses, there are lack of functional AMPA receptors to respond to the release of glutamate. The existence of silent synapses and their contribution to synaptic plasticity are well demonstrated by LTP induction protocols that can quickly recruit postsynaptic AMPA receptors, thus turn silent synapses to functional synapses.^11,12^ In the CA1 region of the hippocampus, activation of NMDA receptors is required for this recruitment.^10^ For intracellular signaling pathways, it is shown that postsynaptic CaMKII plays critical roles in triggering the recruitment of silent synapses.^13,14^ Therefore, this is the most direct evidence for postsynaptic expression of hippocampal CA1 LTP. Due to the limit of electrophysiological experiments, most of these experiments were performed in young hippocampal neurons, and few report of silent synapses in adult hippocampus.

## Silent fibers to silent synapses in pain

In early studies of chronic pain after peripheral injury, it has been reported that some of sensory afferent fibers are inactive or ‘silent’ before the injury.^15–17^ After peripheral injury, such silent afferent fibers became active. It has also been reported that spinal dorsal horn neurons became active after peripheral injury.^17^ However, extracellular recordings of dorsal horn sensory neurons did not allow further investigation of possible synaptic mechanisms. It is also quite possible that such recruitment of neuronal spikes can be due to disinhibition in the spinal dorsal horn.^18,19^ While there are several possible mechanisms contribute to recruitment of silent neurons in spinal cord, silent synapses may be one of strong candidates. Li and Zhuo (1998) reported that many spinal dorsal horn synapses are silent. Postsynaptic protein-protein interaction between AMPA receptors and postsynaptic traffic proteins is required for the recruitment of silent synapses.^20^ There are several subsequent reports, however, suggest that silent synapses may be mainly limited to young neurons in the spinal cord.^21^ One limitation of electrophysiological studies in adult spinal cord is that it is too difficult to clamp sensory neurons using classic whole-cell patch clamp, since many of these dorsal horn neurons send ascending projection to the thalamus (or called spinothalamic cells (STT) cells). It is likely that while the soma potential is at −70 mV, the membrane potentials at distal dendrites may be near positive potentials. In fact, in several early electrophysiological reports, slow EPSPs that are sensitive to the inhibition of NMDA receptors are also detected at the soma resting membrane potential (−65 to −70 mV).^22^ Furthermore, co-application of adenylyl cyclase (AC) activator forskolin and serotonin can recruit new AMPA receptors mediated responses into these pure NMDA synapses.^22^ Thus, the leaking current will be tremendous. Using intracellular recording, Wang et al. (1992) reported that some synapses are completely mediated by pure NMDA receptors. Thus, it is likely that silent synapses may still exist in adult sensory synapses (or filopodia); and such silent ones are difficult to be detected by whole-cell patching recording.

**Figure 1.**
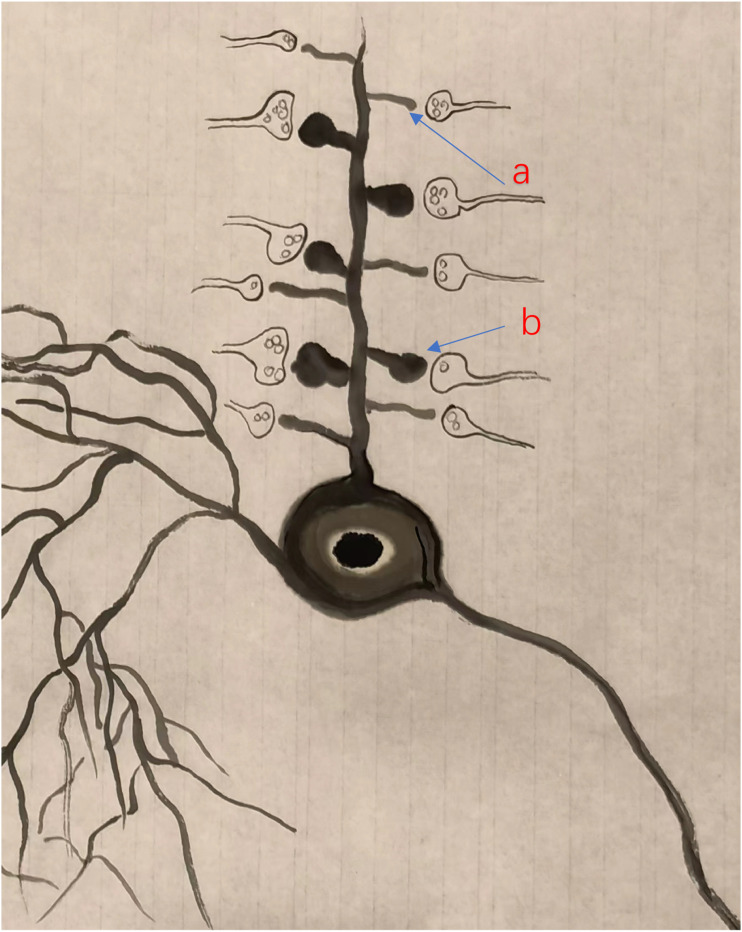
Filopodia is a structure basis for silent synapses in the adult cortex A model of two major forms of spines in a cortical pyramidal cell of an adult mouse. Both thin- and mushroom shaped spines are located at the dendrite of the pyramidal cell.

A recent study in the cortex suggests that filopodia or thin spines are likely silent synapses or synapses containing NMDA receptors^23^ (see Figure 1). In adult spinal cord dorsal, both thin- and mushroom-shaped spines have been reported.^24^ Interestingly, it has been proposed that increase in mushroom-shaped spines (or the recruitment of silent synapses) may contribute to spinal injury-related chronic pain.

## Silent synapses in developmental sensory cortex

Several previous studies have nicely demonstrated that silent synapses exist in developing somatosensory cortex, and it contributes to development-related synaptic potentiation (LTP).^9^ Issac et al. (1997) reported that the loss of ability to undergo LTP is accompanied by the disappearance of silent synapses in adult cortex.^12^ These studies strongly suggest that silent synapses may mainly play roles in central plasticity during neurodevelopment. Interestingly, these silent synapses seem to have less or no role in adult LTP. In the adult cortex where LTP can be detected, it is thought that LTP is mainly achieved by postsynaptic AMPA receptor phosphorylation and/or AMPA receptor insertion; at least for the postsynaptic form of LTP. These consistent results discourage others to search for possible silent synapses in adult cortex, and it is generally believed that silent synapses are more easily to be found in young synapses in the brain.

## Silent synapses (or pure NMDA synapses) in adult anterior cingulate cortex

Previous characterization of silent synapses is mostly by using whole-cell patch-clamp recording technique.^8,10^ When postsynaptic membrane is clamped at resting potentials, voltage-sensitive NMDA receptors are not available to respond to released glutamate. Thus, the minimal stimulation used can be used to mimic one or a few synaptic events. These silent synapses turn not silent when the postsynaptic membrane is depolarized, and NMDA receptor-mediated responses can be observed. However, if we put these silent synapses to the distal dendrites of adult pyramidal cells in the cortex where it is impossible to be inactivated by clamping potentials at the soma, it is possible that these ‘silent’ synapses cannot be detected. In fact, several previous studies using field or intracellular recording method found that some synapses responses at physiological potentials (−65 to 70 mV at the soma) are sensitive to the inhibition of NMDA receptor antagonists.^25^ Furthermore, in freely moving adult mice, it has been reported that there are evoked responses recorded in the ACC by stimulating the other side of ACC that are mainly mediated by NMDA receptors, since AP-5 blocks most of synaptic responses. In adult ACC neurons, NMDA receptor-mediated fEPSPs^26^ and spikes have been reported.^27^ Additional evidence for pure NMDA receptor-mediated responses in the cortex is so-called NMDA spikes. Cortical basal dendrites are a major target for synaptic inputs to pyramidal cells, and there are reports of abundant NMDA receptor-mediated spikes in pyramidal neurons including layer 5 neurons.^28,29^

## Silent synapses in adult (visual) cortex

A recent study by Vardalaki et al. (2022) using different experimental approaches demonstrates that indeed silent synapses exist in deep layer V pyramidal cells of adult visual cortex. They found that there are substantial amount of filopodia (protrusions lacking distinct heads) in adult pyramidal cells. More interestingly, such filopodia are not just limited to layer V pyramidal cells; they can be also found in layer II/III pyramidal cells. Using selective AMPA and NMDA receptor makers, filopodia are found to be exclusively NMDA receptor-positive, and AMPA receptors negative; indicating a possible pure NMDA receptor expressed synapses. Electrophysiological experiments further confirm that these filopodia are indeed silent, pure NMDA synapses. They further found that an LTP induction protocol (spike-timing protocol) is sufficient to unsilence silent synapses. These experiments meet all previously established silent synapses in the hippocampus of young animals and spinal cord, strongly suggest that silent synapses that are sensitive to LTP recruitment exist in adult cortical neurons, and filopodia are a structural substrate for such silent synapses.^23^ These experiments provide strong evidence that filopodia can serve as a marker for silent synapses in the adult brain. Are there filopodia or filopodia-like structures reported in the adult cortex? Future experiments are clearly needed to investigate these possibilities in other areas of the cortex.

## ACC and insular cortex serve as key cortical regions for pain perception

ACC and IC are two key cortical regions for pain perception.^1,3,4,7,30,31^ Neurons in the ACC receive peripheral sensory inputs through thalamic projections.^3,32^ In adult ACC of human and animals, ACC neurons respond to peripheral noxious stimuli, and the neuronal responses are related to the intensity of noxious stimuli. In conscious human brain imaging studies, it has been reported that the intensity of ACC activation is correlated with the intensity of pain unpleasantness. Recent studies have further expanded our understanding of the ACC. Glutamatergic synapses in the ACC, just like those found in the hippocampus, are highly plastic.^1,3^ LTP and long-term depression (LTD) can be found in layer II/II and V pyramidal cells of ACC of adult animals.^1,3^ Both presynaptic and postsynaptic forms of LTP (pre-LTP and post-LTP) have been identified, and post-LTP is activated by NMDA receptors, and the expression is mediated by GluA1 containing AMPA receptors.^1,33,34^ Biochemical and genetic studies suggest that post-LTP is likely mediated by the phosphorylation of AMPA receptor at Ser 845 through cAMP-dependent protein kinase A (PKA).^33^

## Inactive synaptic responses recruited by LTP in the ACC

**Figure 2.**
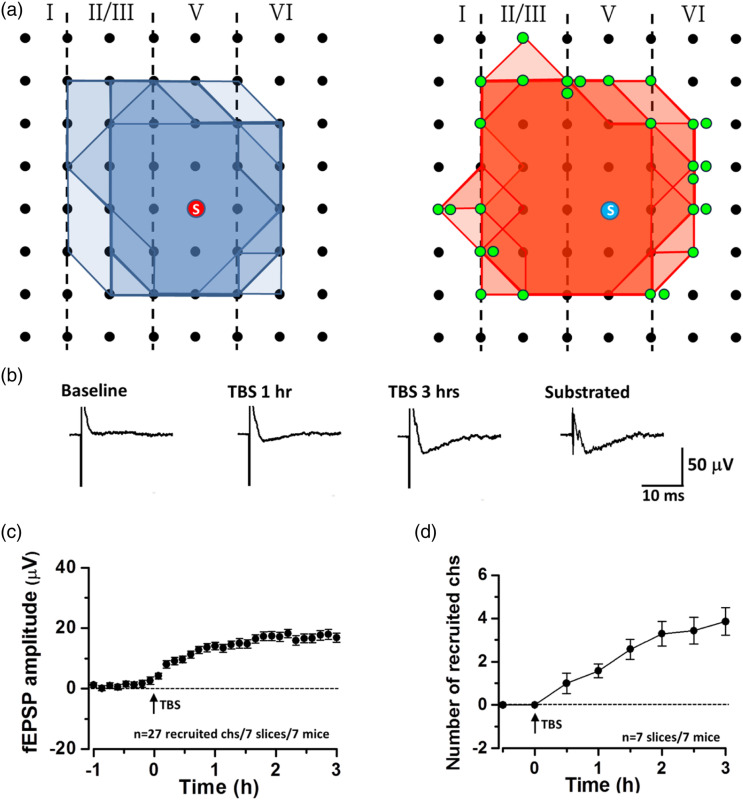
Recruitment of synaptic responses within the ACC after the induction of L-LTP (a) The polygonal diagram showed the baseline area of the activated channels with fEPSP (blue) and the enlarged area after TBS (red). The circled S indicates the stimulation site. The recruited channels were shown as green dots. In some sites, more than one green dot was shown. It means the recruited fEPSP could be observed in the same channel in different slices (*n* = 7 slices/7 mice). (b) The superimposed traces indicate one channel showing recruited fEPSP after TBS. (c) The amplitude of fEPSP was summarized from all recruited channels (*n* = 27 channels from 7 slices/7 mice). (d) The number of recruited channels was summarized after TBS induction. Modified from Chen et al., 2014.

In a recent study of young mice (postnatal 28), it has been reported that silent synapses in the ACC pyramidal neurons that receive thalamic projections.^35^ Due to the complexity of cortical pyramidal neurons in adult mice, it is difficult to have a good space clamp. In our preliminary studies, we have also found filopodia-like structures in pyramidal cells of ACC of adult mice and tree shrews (unpublished observation). Interestingly, recent studies using MED 64 recording channels found that some silent responses can be detected in the ACC.^33–38^ Although field recording with such microelectrodes is difficult to assure the exact origin of electronic source of responses, LTP inducing protocol, however, can clearly recruit active responses from the same sites (Figure 2). A recent study using the combination of single neuron whole-cell patch and MED multiple electrode stimulation revealed that an ACC pyramidal cell is likely receiving different, independent synaptic inputs from each stimulation site.^39^ Considering the high density of synaptic connections in the adult cortical circuit, it is possible that such ‘recruited’ silent responses may be due to the recruitment of silent synapses (filopodia after LTP inductions). Here I propose a hypothesis that such silent responses are actually the results of recruited responses in filopodia or filopodia-like silent synapses. Future studies are clearly needed to examine such possible. Results from filopodia and silent responses in MED studies both consistently suggest that silent synapses do exist in the adult cortex.

## ‘Recruited’ responses are mediated by AMPA receptors

Furthermore, genetic and pharmacological evidence strongly demonstrated that those recruited synaptic responses in silent units are mediated by AMPA receptors, or more precisely, GluA1-containing receptors.^33,36^ Chen et al. (2014) reported that TBS-induced late-phase LTP (L-LTP) in the ACC of Fmr1 WT mice were able to recruit silent responses that were inactive during baseline recordings (see Figure 2). These newly established responses are stable once established and persisted for the same period of time as those potentiated active channels.^36^ Song et al. (2018) further confirmed the recruitment of silent responses by TBS-induced L-LTP in the ACC and found that the recruitment of silent responses was blocked in GluA1 845 knock-in mice, indicating that GluA1 is required for such recruitment. This result is in parallel with blocked LTP of active channels in the same slices. These results consistently suggest that there are at least two populations of individual synapses that undergo LTP in the ACC. The first one is active synapses, the potentiation in these synapses is likely due to modification of existing AMPA receptors by intracellular signaling pathways. The second one is silent synapses where there is no functional AMPA receptor before LTP induction. The recruitment of GluA1-containing receptors is likely to occur after LTP. Once it is induced, these new synapses are stable, and mediated increased synaptic responses in the ACC.

## Inactive responses can also be recruited chemically

In addition to LTP, these silent responses can be also recruited chemically by different signaling peptides.^37,40^ Li et al. (2019) reported that bath application of calcitonin gene-related peptide (CGRP) can recruit silent responses in the ACC of adult mice, and this effect is likely mediated through CGRP receptors (Figure 4). Furthermore, the activation of NMDA receptor is also required for CGRP-induced recruitment. Brain-derived neurotrophic factor (BDNF) is known to play important roles in cortical plasticity. On activity-dependent manner, BDNF can be released from neurons. In addition, there are reports of glia-source. By bath application of BDNF, Miao et al. (2021) reported in the ACC that BDNF recruits silent responses and such recruited responses persistent for at least 3 hrs. A persistent and long-lasting nature of active responses fits silent synapses recruited by LTP really well. Furthermore, Zhou et al. (2023) found that the recruitment of silent synapses by ACC LTP is age-related. In middle-aged mice, there is a reduction of number of silent responses recruited by LTP, in parallel with the reduction of LTP in the active synapses.^38^ These results indicate that aging process may affect the ability of LTP to recruit silent synapses in the brain. Zhou et al. (2023) further suggest that the reduced level of BDNF may be one of causes, since BDNF or a selective TrkB receptor agonist can rescue this effect in middle-aged mice.

**Figure 3.**
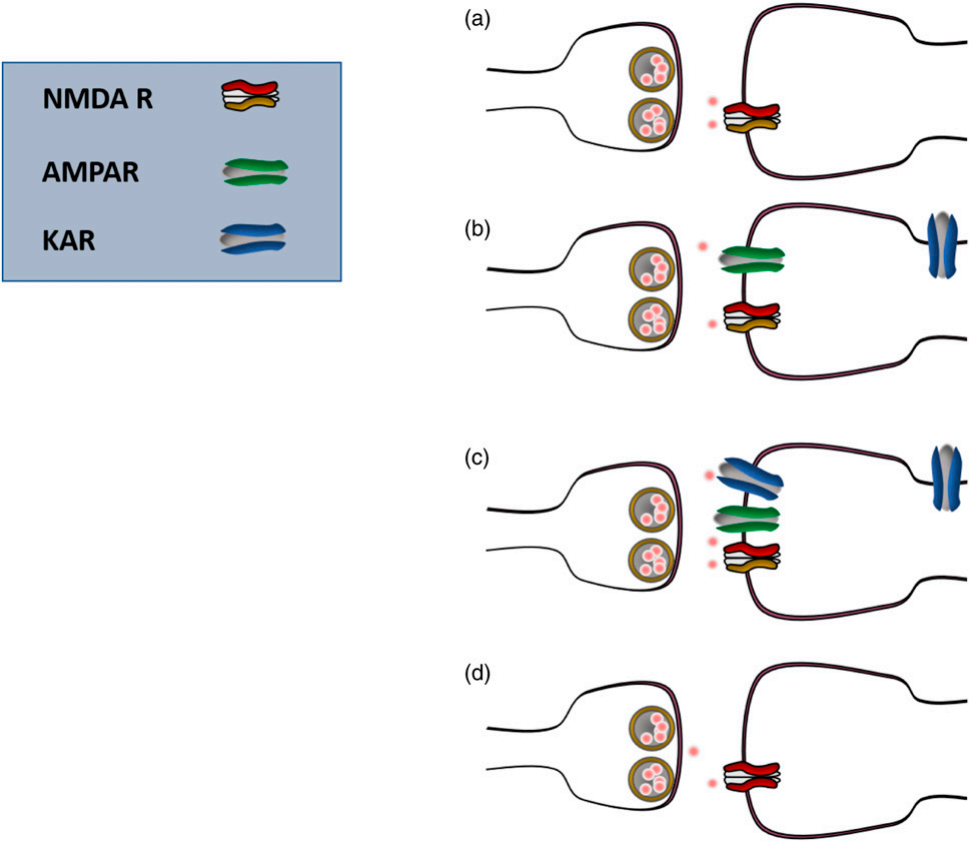
Glutamatergic synapses in the adult cortex are heterogenous. There are at least 4 different types of synapses in adult ACC neurons: silent (pure NMDA receptor) synapses (a); pure AMPA receptor-containing synapses (b), mixed AMPA and KA synapses (c), and functional, pure NMDA receptor synapses (d).

In summary, it is likely that glutamatergic synapses in adult ACC are heterogenous (Figure 3). In addition to two possible functional synapses that are pure AMPA and AMPA and kainate (KA) receptor mixed synapses, there are silent synapses and synapses containing functional NMDA receptors. In physiological condition, the functional NMDA receptors are likely located at distal dendrites or other sites where the resting membrane potentials are at the level that allow NMDA receptors to be activated. This type of NMDA receptor-containing synapses is likely the contributor for well known central side effects of NMDA receptor antagonists in physiological in vivo conditions.

## Intracellular pathways for the recruitment of silent synapses in the cortex

**Figure 4.**
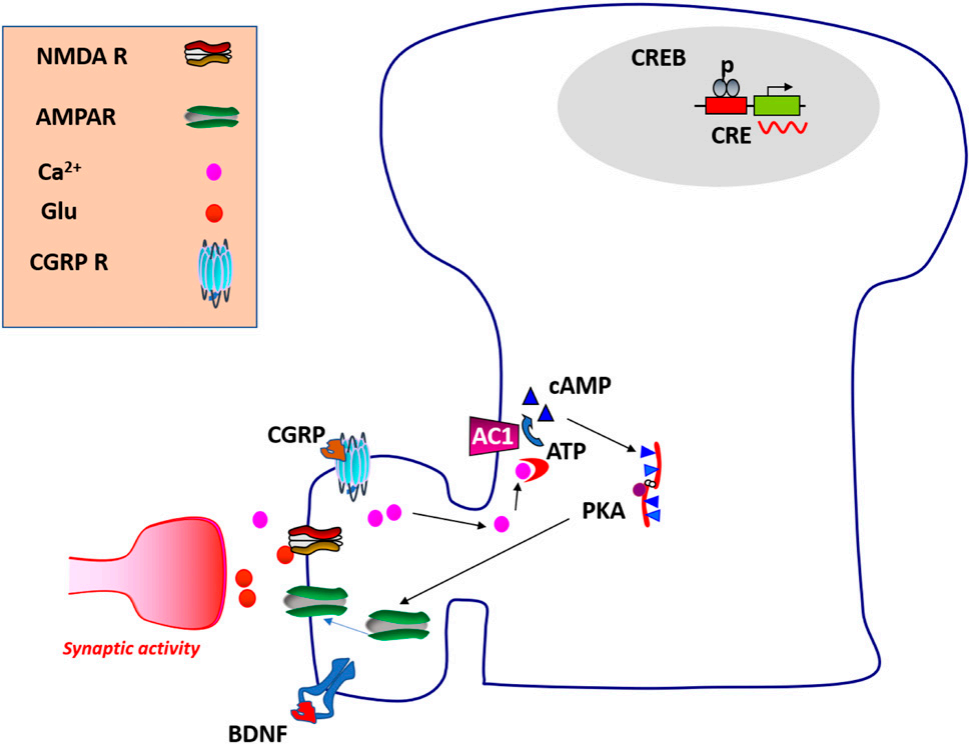
Proposed signaling pathways for the recruitment of silent synapses in the cortex. A diagram showing the intracellular signaling pathways for the recruitment of silent synapses in adult synaptic spines. Postsynaptic NMDA receptors are activated by an activity-dependent manner. Subsequent calcium influx then binds to intracellular calmodulin, and then leads to activation of neuronal selective AC1. cAMP as a second messenger triggers PKA activity. GluA1 subunit-containing AMPA receptor is regulated by two different PKA phosphorylation sites, Serine 845 and 834. Serine 845 is required for the recruitment of silent responses. In addition to NMDA receptors, G protein-coupled CGRP receptors may also contribute to the recruitment. TrKB receptor, a receptor that binds to BDNF, can contribute to the recruitment.

Previous studies of intracellular mechanism of ACC LTP provide a possible explanation for the basic mechanisms of the recruitment of silent synapses. It is clear that the recruitment of silent synapses serves as a key mechanism for a postsynaptic form of LTP in the ACC, in addition to the potentiation of active, existed AMPA receptor-mediated responses. It is likely that calcium-stimulated ACs are important for the recruitment of silent synapses in pyramidal cells (Figure 4). Genetic and pharmacological findings indicate that AC1 or AC1 activity is required for the recruitment of silent responses in the ACC.^33–37^ Postsynaptic increases in Ca^2+^ triggered by activation of NMDA receptors or other types of signaling sources.^1^ Among several types of ACs, AC1 is the most sensitive form to Ca^2+^. Increases in 2^nd^ messenger cAMP then leads to activation of PKA, and the phosphorylation of AMPA receptors by PKA play important roles in the potentiation of AMPA receptor-mediated responses^33^ as well as the recruitment of silent responses.^33,36^ In addition, PKMzeta (PKMζ) activity maybe also required. It is likely that these signaling pathways are shared by silent synapses and the potentiation of AMPA receptors in the ACC. It is important for the future to investigate if other signaling molecules may also contribute to the recruitment of silent synapses as well as the potentiation of AMPA receptor-mediated responses. Fragile X mental retardation protein (FMRP), a key intracellular signaling molecule is found to be important as well.^36^

## Functional implications for adult silent synapses in chronic pain and emotion

Cortical excitation or potentiation has been proposed to contribute to chronic pain. More specially, two major forms of LTPs have been found in the ACC and IC. Pre-LTP is thought to contribute to injury-related emotional changes, post-LTP plays important roles in chronic pain and injury-related behavioral hyperalgesia and allodynia. Silent synapses (or filopodia) add new mechanisms for critical potentiation/excitation. Furthermore, the recruitment of silent synapses may lay the basis for spreading the excitation within the ACC circuit. Such spreading of excitation is likely limited.

In addition to chronic pain, ACC neurons have also been indicated in fear memory.^1,7,41,42,43^ Postsynaptic potentiation of AMPA receptor-mediated responses has been reported after trace fear training.^43^ Postsynaptic increased level of AMPA receptors on the membrane has been reported in the ACC. The recruitment of silent synapses may serve as one of key mechanisms to fear-induced LTP in the ACC. Functionally, these silent synapses may contribute to the induction and expression of fear memory.

ACC pyramidal cells in deep layers have been reported to project to the spinal cord dorsal horn and exert facilitatory modulation of spinal nociceptive transmission.^44,45^ Facilitated responses of dorsal horn neurons may contribute to behavioral hyperalgesia and allodynia in case of chronic pain. Increased postsynaptic responses of ACC pyramidal cells after LTP may enhance such top-down modulation of spinal sensory transmission. Silent synapses in the deep pyramidal cells may thus contribute to top-down descending facilitation.

In addition to pain, fear and sensory modulation, ACC has also been indicated in itch.^46^ In case of chronic itch, it is likely that the recruitment of silent synapses in the ACC may contribute to long-lasting itch.

At the spinal cord level, the recruitment of silent synapses may serve as a key synaptic mechanism for endogenous descending facilitatory modulation from supraspinal structures such as the brain stem and cortex.^45,47^

## Conclusion and future directions

Recent evidence more and more suggest that silent synapses are unlikely limited to the developmental stage. Previous studies that missed these synapses are likely due to the technical limitation, and distal dendrite location. Silent synapses (0 to 1) together with postsynaptic receptor modification (1 to 1x) offer unique distinct mechanisms for synaptic potentiation in the ACC. Such potentiation not only happens at existing connected circuits, but also can recruit new connections or ‘silent’ connections through silent synapses. Future studies are clearly needed to investigate if these two mechanisms may share exact intracellular signaling pathways or if any distinct signaling molecules may be involved. For physiological and pathological significance of silent synapses, it is important to understand possible different mechanisms or molecules that may be involved, and this new information may help us to find better treatment for patients with chronic pain, depression and other mental diseases.
